# Supplementary material for: Walking and rolling of crystals induced thermally by phase transition
Source: Nat Commun. 2018 Feb 7;9:538. doi: 10.1038/s41467-017-02549-2 (PMC5803265; doi:10.1038/s41467-017-02549-2)
Supplement: Supplementary file 1 — Supplementary Information [file 41467_2017_2549_MOESM1_ESM.pdf]

Supplementary Information

Walking and rolling of crystals induced thermally by phase transition

Takuya Taniguchi,[1] Haruki Sugiyama,[2] Hidehiro Uekusa,[2] Motoo Shiro,[3] Toru Asahi,[1,3] Hideko Koshima[3*]

[1] Department of Advanced Science and Engineering, Graduate School of Advanced Science and Engineering, Waseda University, 3-4-1 Okubo, Shinjuku-ku, Tokyo 169-8555, Japan

[2] Department of Chemistry and Materials Science, Tokyo Institute of Technology, Ookayama 2-12-1, Meguro-ku, Tokyo 152-8551, Japan

[3] Research Organization for Nano & Life Innovation, Waseda University, 513 Wasedatsurumaki-cho, Shinjuku-ku, Tokyo 162-0041, Japan

* Correspondence to hkoshima@aoni.waseda.jp

Supplementary Figures 1–13 page S2

Supplementary Tables 1 and 2 S15

Supplementary References S17

Supplementary Figures

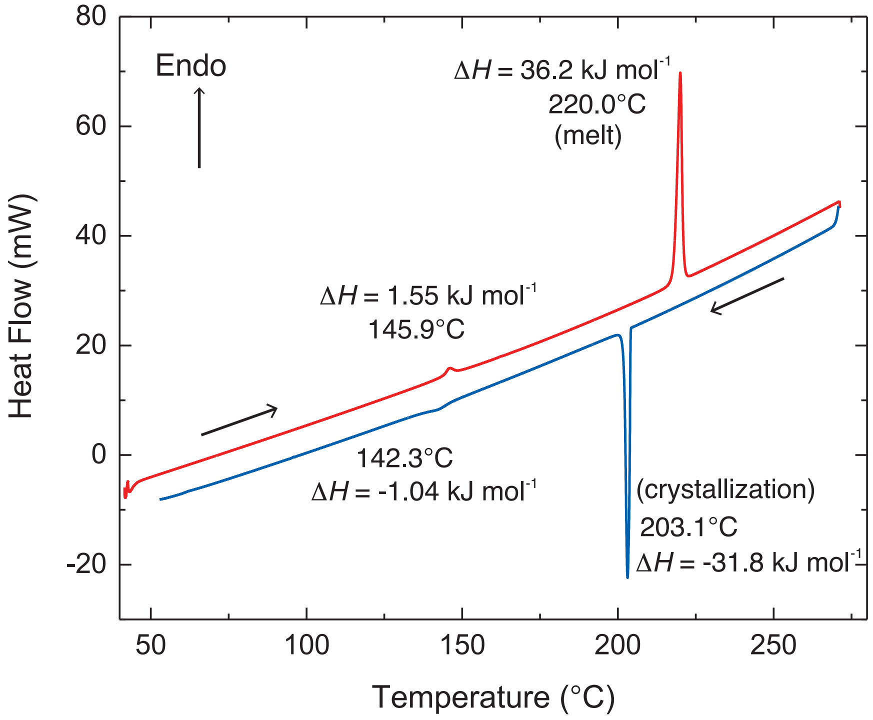

Supplementary Figure 1. **DSC measurement of β crystals of *trans*-(*S*)-1**.
Differential scanning calorimetry (DSC) curve measured in the temperature range of 50–270°C at a rate of 10°C min^{-1} on heating and subsequent cooling. Red and blue lines indicate heating and cooling, respectively.

On heating, the DSC curve showed a small endothermic peak of a phase transition at 145.9°C and a larger endothermic melting peak at 220.0°C. On subsequent cooling, the DSC curve had a large exothermic peak of crystallization at 203.1°C and a small exothermic peak of a phase transition at 142.3°C, showing that the phase transition occurred even after melting and crystallization.

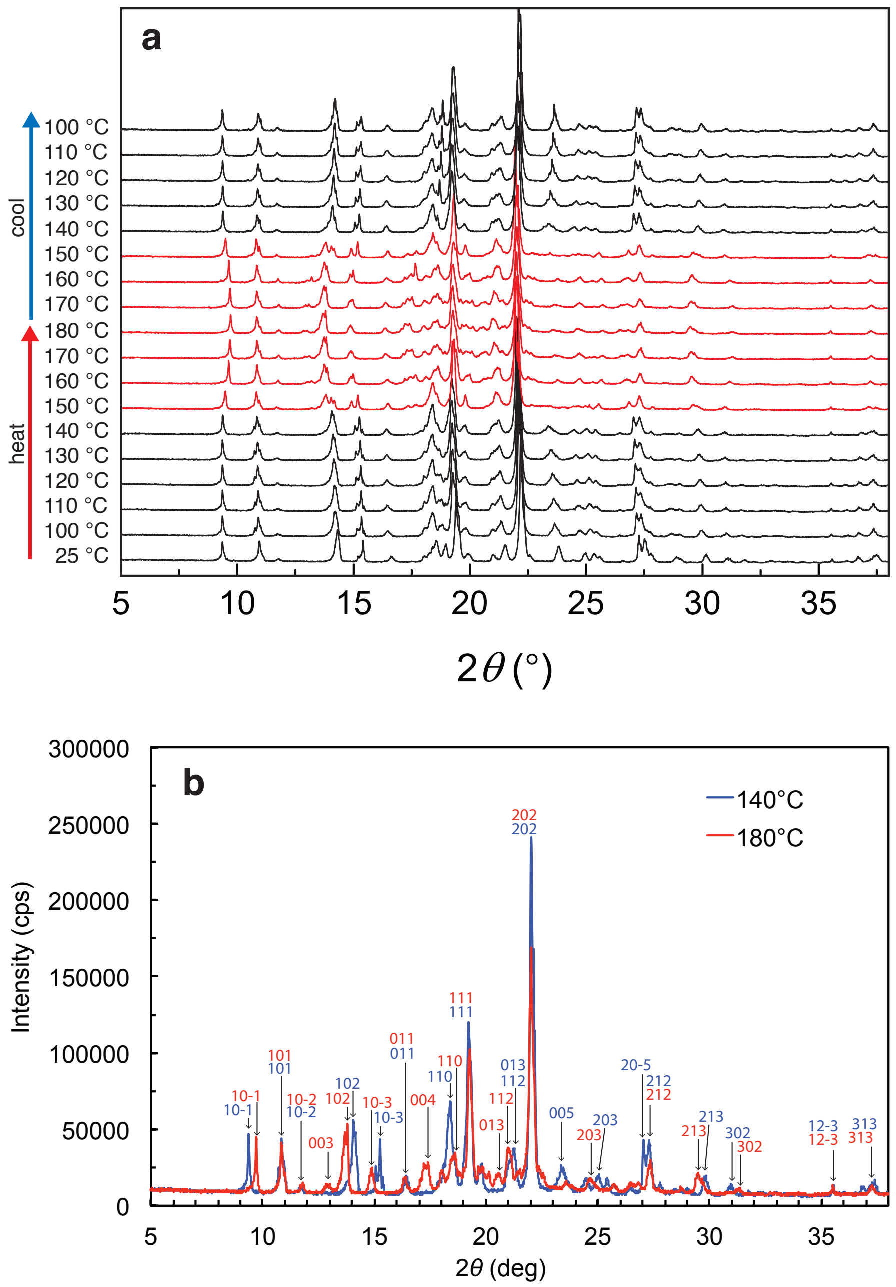

Supplementary Figure 2. **Powder XRD profiles at variant temperatures**.

a, Powder X-ray diffraction (XRD) patterns at 25°C and over the temperature range of 100–180°C at 10°C intervals on heating and then cooling. XRD patterns shown in black and red lines indicate the β and γ crystals, respectively. **b**, Comparison of XRD patterns of the β phase at 140°C and the γ phase at 180°C.

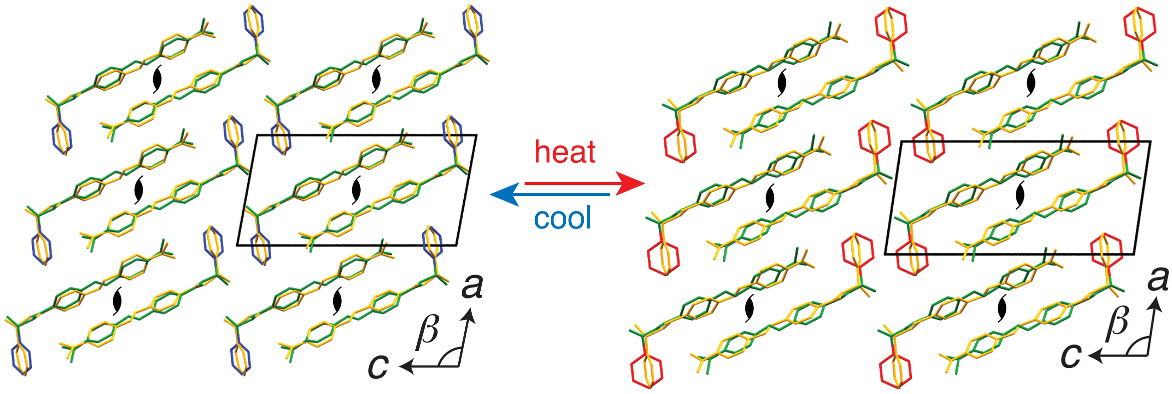

Supplementary Figure 3. **Molecular arrangements on the (010) face before and after phase transition**.

Two-fold helical structures are formed along the *b* axis at 125 and 160°C. Conformers A and B are shown in green and yellow, respectively. Hydrogen atoms are omitted for clarity.

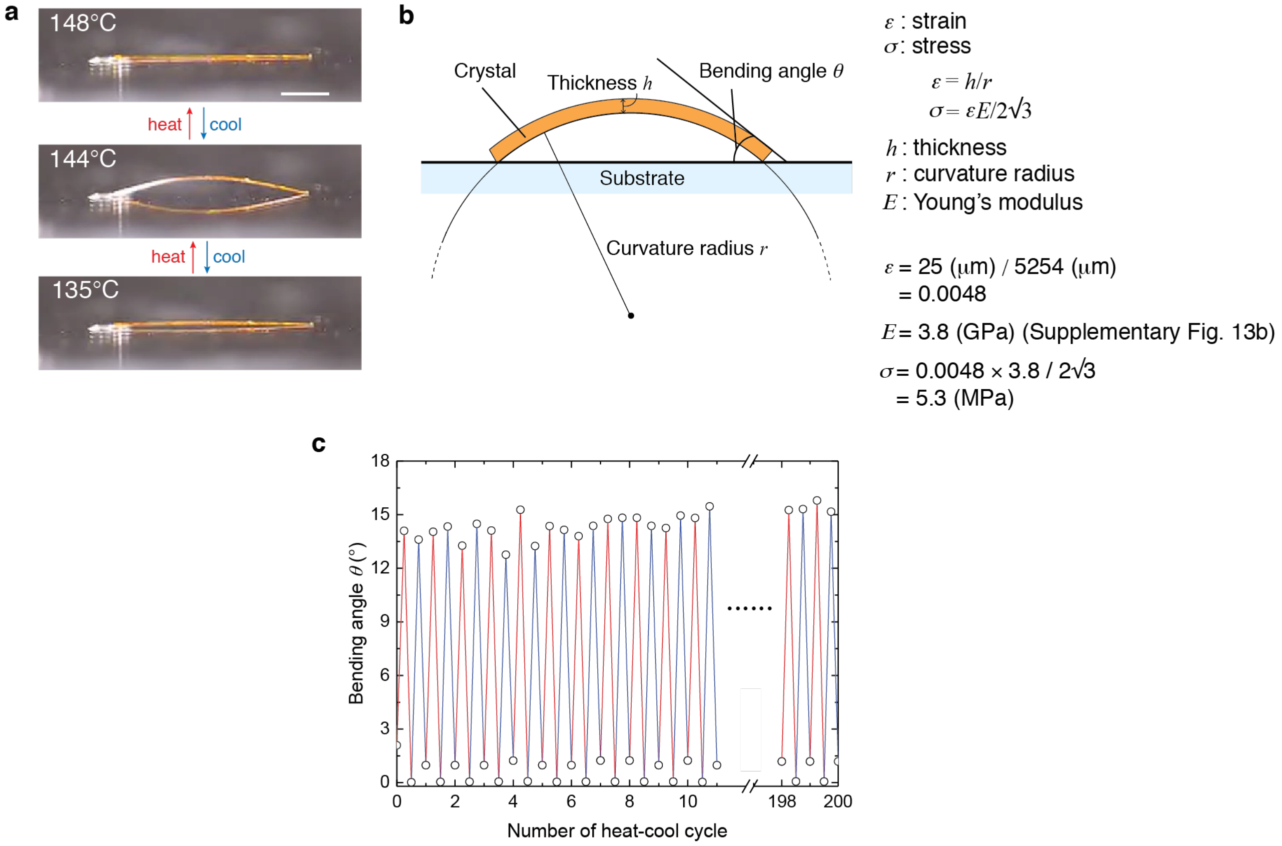

Supplementary Figure 4. **Bending of a thin, long plate-like crystal and mechanical properties of bending**. **a**, Bending motion of a *trans*-(*S*)-**1** crystal (2100 × 50 × 25 μm^3); the left tip of which was fixed with glue to prevent locomotion (Supplementary Movie 4). The motion was recorded with an optical microscope from the side. The mirror image was reflected on the glass surface. The scale bar is 500 μm. **b**, Definition of bending angle θ and calculation of strain and stress. The value of stress (5.3 MPa) was calculated by using the Young's modulus (3.8 GPa) shown in Supplementary Fig. 13b. **c**, Reversibility of bending motion. When the bending reached a maximum in each process of heating or cooling, the bending angle θ was calculated using the movie analysis software ImageJ[1].

The longer, thinner crystal began to bend at 140°C on heating, and reached a maximum bending angle, θ = 15°, at 143–144°C. Then, the crystal became straight above 145°C due to the completion of the phase transition to the γ phase. On subsequent cooling, the crystal bent suddenly with a maximum bending angle, θ = 15°, at 141°C, and then returned to a straight form at 136°C. The slight temperature difference in bending between heating and cooling may be due to the thermal hysteresis in the reversible phase transition.

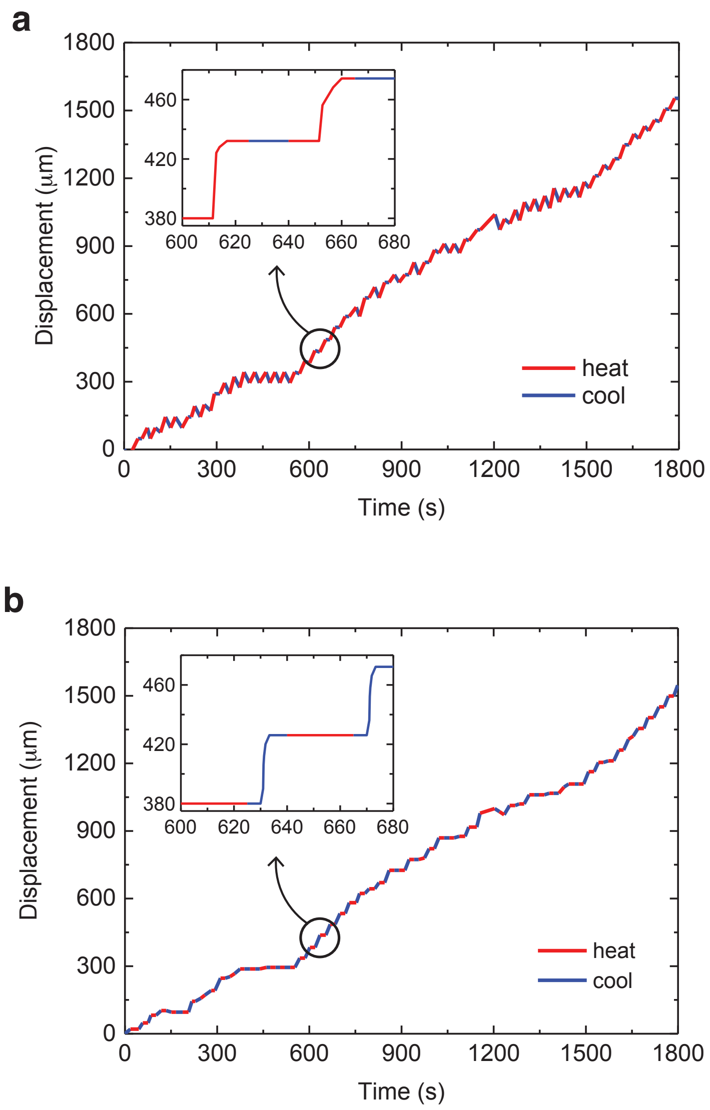

Supplementary Figure 5. **Time dependence of inchworm-like walking on a silanized glass**. **a,b**, Distances of left (**a**) and right (**b**) edges from initial positions of the crystal shown in Fig. 4 in the main text. Displacements were measured from Supplementary Movie 5. Duration of heating and cooling was extracted also from Supplementary Movie 5 corresponding to the crystal motion.

On heating, the thicker left edge tended to move to the right, and the thinner right edge tended to stop. On cooling, the thicker left edge tended to stop or move slightly to the left, and the thinner right edge tended to move to the right.

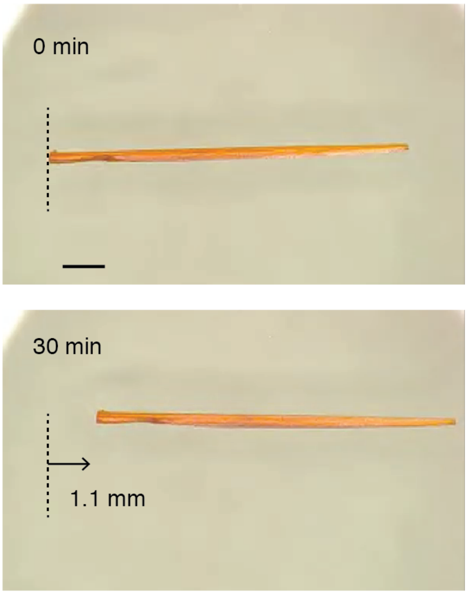

Supplementary Figure 6. **Inchworm-like walking on a glass without surface treatment.**
On a glass without surface treatment, the same crystal in Fig. 4 in the main text moved 1.1 mm to the right direction in 30 min (walking speed 2.2 mm h^{-1}) under the same heating and cooling rate in Fig. 4a, which was slower than the walking speed 3.0 mm h^{-1} on a silanized glass. The scale bar is 1 mm.

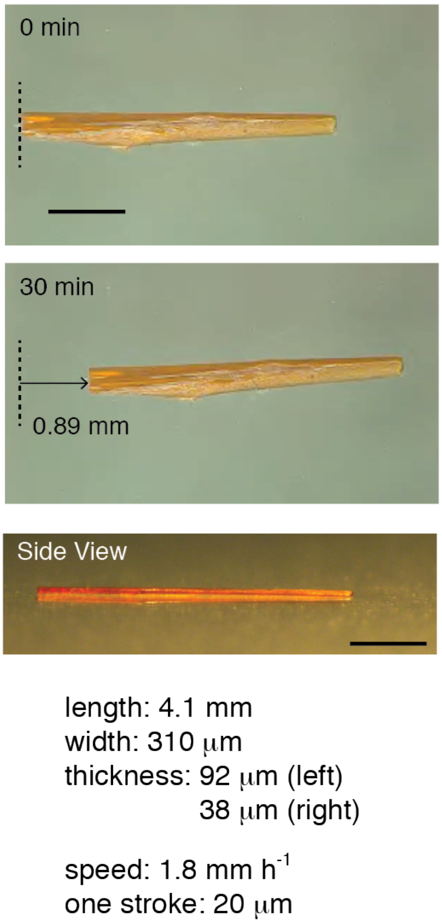

Supplementary Figure 7. **Inchworm-like walking of plate-like crystal with thickness gradient.** A shorter plate-like crystal with a thickness gradient was heated and cooled on a silanized glass with the same temperature rate in Fig. 3a in the main text. The crystal moved 0.89 mm in 30 min (walking speed 1.8 mm h^{-1}). The average one stroke was 20 μm. The scale bars are 1 mm.

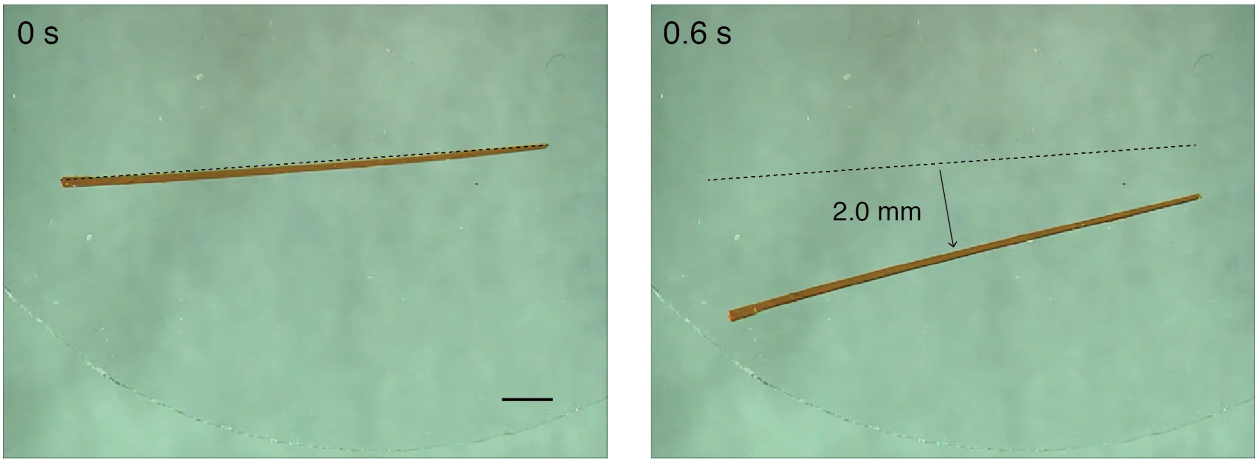

Supplementary Figure 8. **Fast rolling locomotion of a long, thin plate-like crystal on cooling**. This is the same crystal in Fig. 5 in the main text. When the right and left of the crystal were reversed on a silanized glass with the same temperature condition, the crystal rolled to the lower direction, which was the opposite direction to that in Fig. 5. The result suggests that the direction of rolling locomotion depends on the unsymmetrical crystal shape. The scale bar is 1 mm.

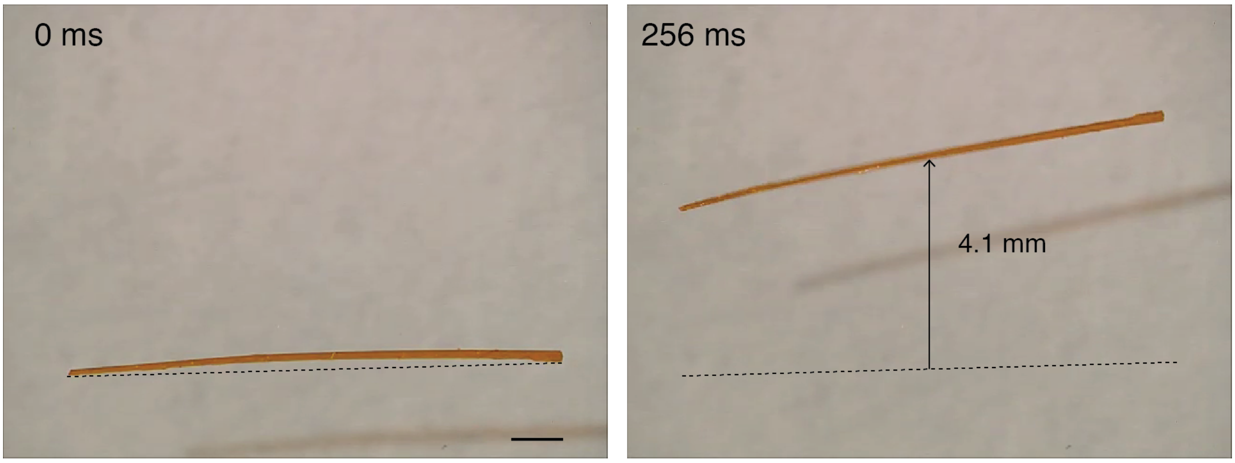

Supplementary Figure 9. **Fast rolling locomotion of a long, thin plate-like crystal on a glass without surface treatment upon heating**.

On a glass without surface treatment, the same crystal in Fig. 5 in the main text flipped repeatedly and moved 4.1 mm in 256 ms (rolling speed: 16 mm s^{-1}). The scale bar is 1 mm.

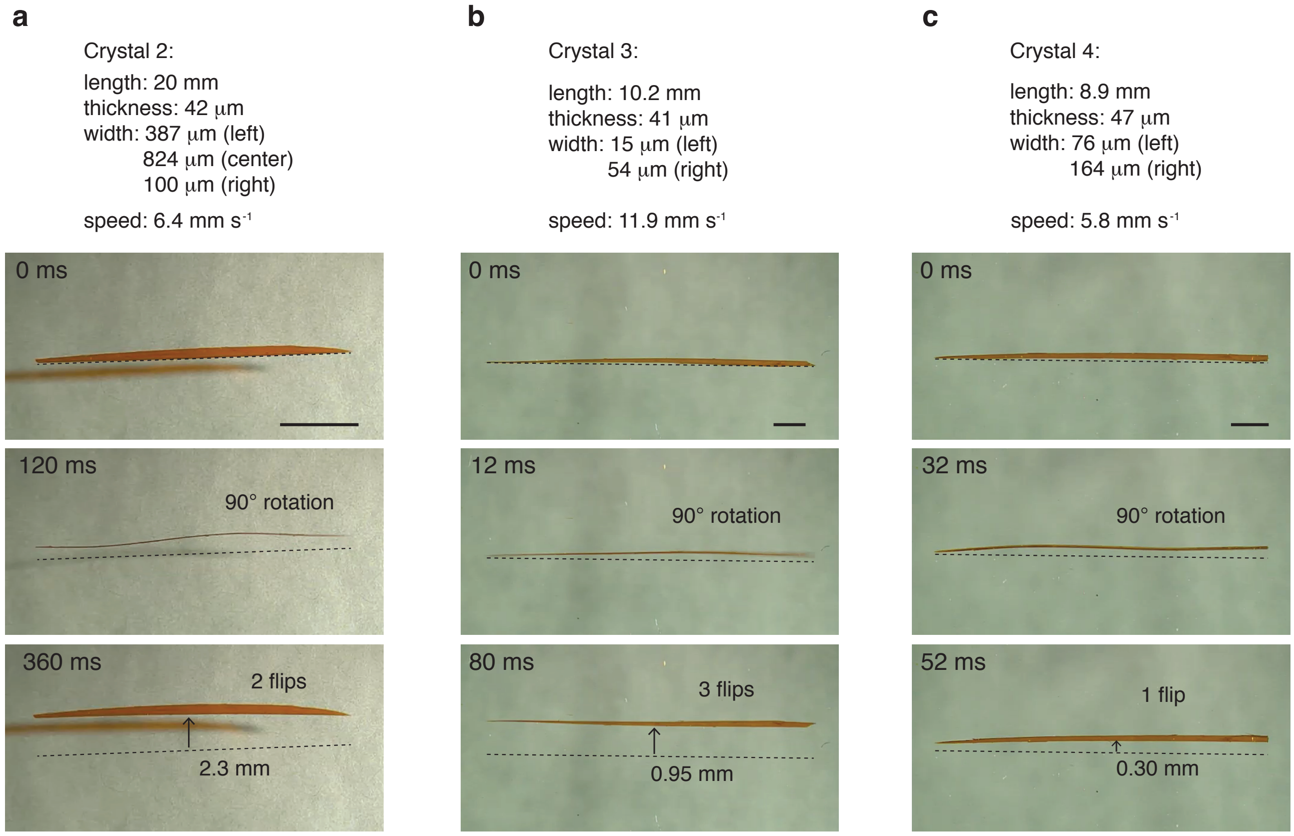

Supplementary Figure 10. **Fast rolling locomotion of long, thin plate-like crystals with unsymmetrical shape on cooling**.

Dotted lines indicate the initial position. Each crystal was numbered as the reference for Supplementary Fig. 12. Crystal 2 was observed on a glass without surface treatment, and crystals 3 and 4 were observed on silanized glass plates. The longer, wider crystal 2 needed longer time to cause the flipping than those of the shorter, narrower crystals 3 and 4. The crystals flipped to the direction where the angle of the crystal corner is obtuse angle. The scale bars are 5 mm in **a**, and 1 mm in **b** and **c**.

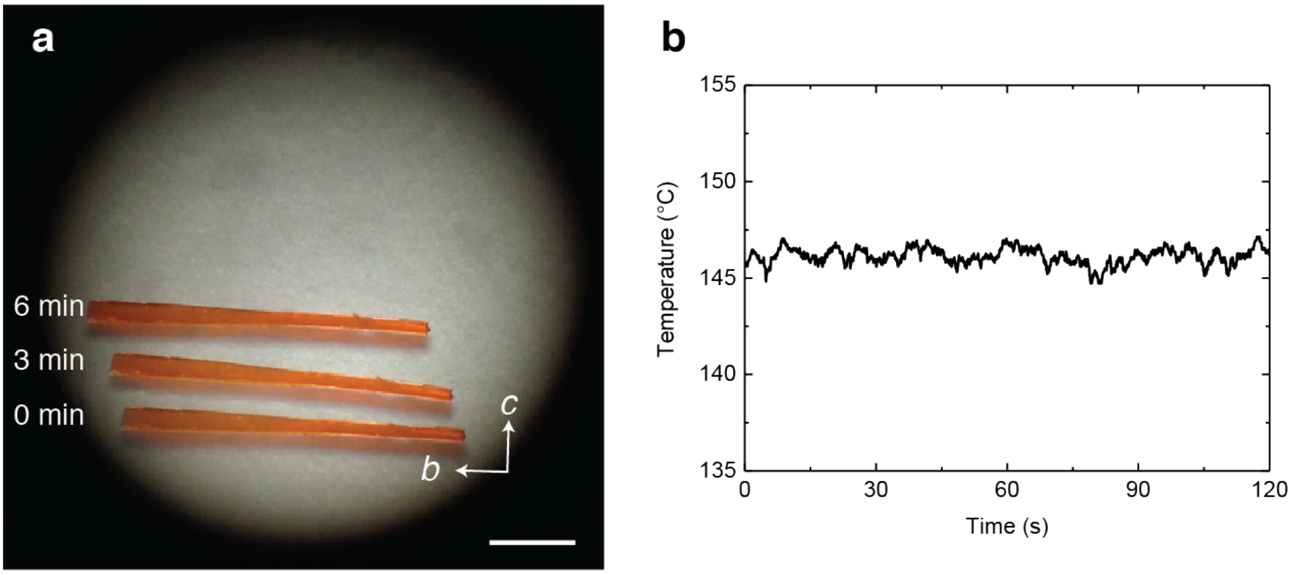

Supplementary Figure 11. **Slow locomotion of a short, thick plate-like crystal without rolling**. **a**, Locomotion of a crystal (3990 × 240 × 108 μm^3) on a silanized glass by repeated heating and cooling near the transition point, 145°C (Supplementary Movie 10). The scale bar is 1 mm. **b**, Temperature fluctuation of the silanized glass, measured with an IR thermometer.

The crystal moved very slowly in a direction almost perpendicular to the long axis, with repeating the small bending and straightening; the moving distance was only 1.5 mm after 6 min (moving speed 0.0042 mm s^{-1}).

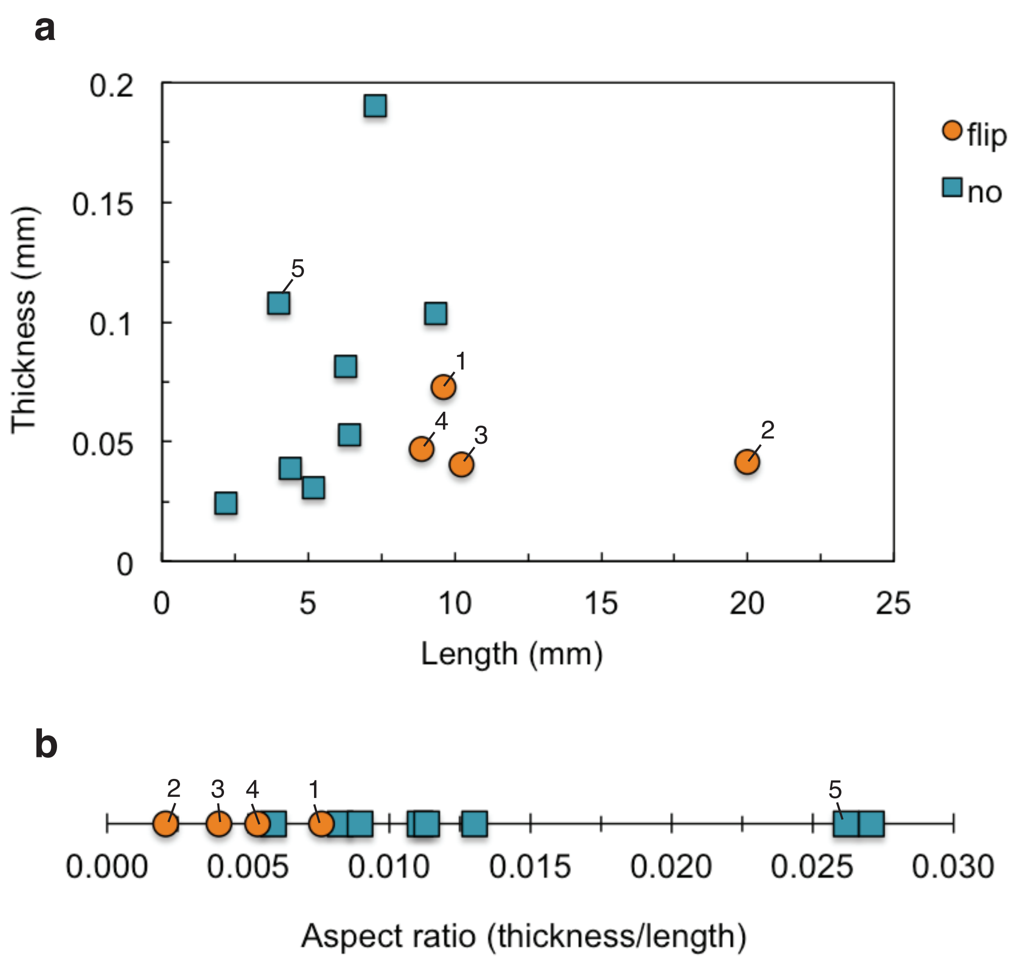

Supplementary Figure 12. Relationship between crystal shape (length and thickness) and locomotion with and without rolling (**a**), **and that between aspect ratio (thickness/length) and locomotion** (**b**).

Numbers in the graph indicate different crystals.

1: the crystal in Fig. 5 in the main text and Supplementary Fig. 8 and 9

2,3,4: the crystals in Supplementary Fig. 10

5: the crystal in Supplementary Fig. 11

The crystals of aspect ratio (thickness/length) 0.002~0.008 caused flipping. In contrast, the crystals of that 0.006~0.027 did not flip.

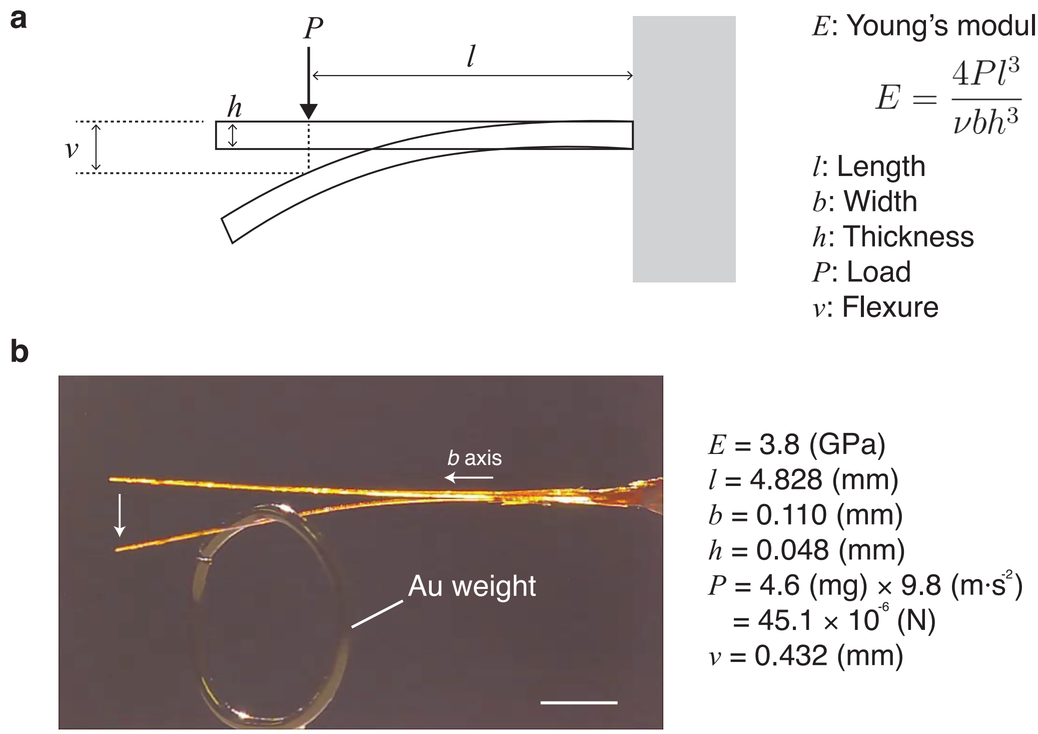

Supplementary Figure 13. **Cantilever-bending test to measure Young's modulus of the β-crystal**.

a, Schematic illustration of cantilever-bending test[2]. **b**, Experimentally obtained data. A long plate-like crystal of *trans*-(*S*)-**1** (6889 × 110 × 48 μm^3) was fixed with glue at the right tip. A gold weight (4.6 mg) was placed manually on the (100) face of the crystal, and flexure was then measured. The picture shows before and after applying the load. The scale bar is 1 mm.

The value of Young's modulus (3.8 GPa) obtained here was used for the calculation of the stress (5.3 MPa) shown in Supplementary Fig. 4b.

Supplementary Tables

Supplementary Table 1. Crystallographic parameters of the β and γ crystals of *trans*-(*S*)-1.

Temperature (°C)	-100 (β)	20 (β)	100 (β)	125 (β)	160 (γ)
Empirical formula	$C_{23}H_{24}N_4O$	$C_{23}H_{24}N_4O$	$C_{23}H_{24}N_4O$	$C_{23}H_{24}N_4O$	$C_{23}H_{24}N_4O$
Formula weight	372.47	372.47	372.47	372.47	372.47
Crystal system	Monoclinic	Monoclinic	Monoclinic	Monoclinic	Monoclinic
Space group	$P2_1$	$P2_1$	$P2_1$	$P2_1$	$P2_1$
a (Å)	9.7488(6)	9.7660(3)	9.7655(6)	9.7523(16)	9.345(7)
b (Å)	5.3916(3)	5.5160(2)	5.5633(4)	5.5687(9)	5.552(4)
c (Å)	18.8176(11)	18.9256(6)	19.0436(12)	19.131(3)	20.474(15)
β (°)	100.605(7)	100.7202(17)	100.547(2)	100.432(6)	98.416(19)
V (Å^3)	972.19(10)	1001.70(6)	1017.12(11)	1021.8(3)	1050.9(13)
Z	2	2	2	2	2
ρ_{calc} (g·cm^{-3})	1.272	1.235	1.216	1.211	1.177
R_1 [$I > 2\sigma(I)$]	0.0694	0.0365	0.1051	0.0681	0.1190
wR_2 [$I > 2\sigma(I)$]	0.1552	0.0876	0.2999	0.1864	0.3059
GOF	1.062	1.207	1.159	0.886	0.743

Supplementary Table 2. **Occupancy, dihedral angle between the azobenzene plane and the phenyl ring of the side chain, and intermolecular hydrogen bond distance of each conformer of the disordered molecule**.

Temperature (°C)	-100 (β)		20 (β)		100 (β)		125 (β)		160 (γ)	
	A	B	A	B	A	B	A	B	A	B
Occupancy (%)	100	0	66.6	33.4	59	41	61.7	38.3	44.4	55.6
Dihedral angle (°)	82.38	-	84.09	84.85	82.74	86.11	81.03	87.93	56.17	82.88
NH···O=C hydrogen bond distance (Å)	2.379	-	2.557	2.499	2.621	2.546	2.603	2.728	2.596	2.664

Because the azobenzene skeleton is almost planar, the dihedral angle between the azobenzene plane and the phenyl ring of the side chain in each conformer could be calculated. All non-H atoms of Ph-N=N-Ph were picked to form an azobenzene plane by least-square mean calculation, programmed in Mercury. All C-atoms of the phenyl ring of the side chain were also selected to create another plane. Then, the dihedral angle between the two planes was calculated for each conformer.

Supplementary References

1. Schneider, C. A., Rasband, W. S. & Eliceiri, K. W. NIH Image to ImageJ: 25 years of image analysis. *Nature Methods* **9**, 671-675 (2012).
2. Murakami, T., Yoshimura, T. & Hikosaka, H. *Statics of Structures (1)*; Corona Publishing: Tokyo, Japan (1983).
