# Supplementary material for: Walking and rolling of crystals induced thermally by phase transition
Source: Nat Commun. 2018 Feb 7;9:538. doi: 10.1038/s41467-017-02549-2 (PMC5803265; doi:10.1038/s41467-017-02549-2)
Supplement: Supplementary file 3 — Description of Additional Supplementary Information [file 41467_2017_2549_MOESM3_ESM.pdf]

Description of Additional Supplementary Files

File Name: Supplementary Movie 1
Description: Shape change of a plate-like crystal of trans-(S)-1 viewed from the (100) top face on heating and then cooling (played at 20× speed).

File Name: Supplementary Movie 2
Description: Shape change of the crystal of trans-(S)-1 viewed from the (010) cross-section face on heating and then cooling (played at 20× speed).

File Name: Supplementary Movie 3
Description: Shape change of the crystal of trans-(S)-1 viewed from the (001) side face on heating and then cooling (played at 20× speed).

File Name: Supplementary Movie 4
Description: Bending motion of a long, thin crystal of trans-(S)-1, the left edge of which was fixed with glue, on heating and then cooling at a rate of 1°C s-1 over the temperature range of 135–148°C (played at 2× speed).

File Name: Supplementary Movie 5
Description: Inchworm-like walk to longitudinal direction of a plate-like crystal with a thickness gradient during on repeated heating and cooling (played at 50× speed).

File Name: Supplementary Movie 6
Description: Surface temperature distribution of the plate-like crystal with the thickness gradient on heating and then cooling measured by an IR thermography camera, and inchworm-like walk observed simultaneously by a microscopy (played at 4× speed).

File Name: Supplementary Movie 7
Description: Fast flipping running of a long, thin plate-like crystal with a width gradient on heating (real time).

File Name: Supplementary Movie 8
Description: Slow-motion-movie of flipping running of the same crystal as Supplementary Movie 7 on cooling (played at 0.03× speed).

File Name: Supplementary Movie 9
Description: Slow-motion movie taken from slant upper side view of flipping running of the same crystal as Supplementary Movie 7 on cooling (played at 0.03× speed).

File Name: Supplementary Movie 10
Description: Slow locomotion without flipping when the same crystal as Supplementary Fig. 9 was repeatedly heated and cooled near the transition point 145°C on a silanized glass plate (played at 10× speed).

File Name: Supplementary Data 1
Description: Crystal structure of trans-(S)-1 at -100°C.

File Name: Supplementary Data 2
Description: Crystal structure of trans-(S)-1 at 20°C.

File Name: Supplementary Data 3
Description: Crystal structure of trans-(S)-1 at 100°C.

File Name: Supplementary Data 4
Description: Crystal structure of trans-(S)-1 at 125°C.

File Name: Supplementary Data 5
Description: Crystal structure of trans-(S)-1 at 160°C.
